# Draft genome sequence of a Lactiplantibacillus pentosus strain isolated from traditionally fermented rice

**DOI:** 10.1099/acmi.0.000796.v3

**Published:** 2024-10-03

**Authors:** Athira Cheruvari, Rajagopal Kammara

**Affiliations:** 1 Department of Biochemistry, Central Food Technological Research Institute, Mysore 570020, India; 2 Academy of Scientific and Innovative Research, Ghaziabad 201002, India

**Keywords:** fermented foods, *Lactiplantibacillus pentosus*, RAST annotation, whole genome sequencing

## Abstract

*Lactiplantibacillus pentosus* is a probiotic bacterium reported to be present in various fermented foods, such as fermented olives, and it significantly influences human health. The present study concerns a lactic acid bacterial strain designated *L. pentosus* krglsrbmofpi2, isolated from traditional fermented rice, and which has been shown to have an assortment of beneficial attributes. Using Illumina technologies, we have sequenced and investigated the whole genome sequence of * L. pentosus* krglsrbmofpi2 to understand its functionality and safety. The chromosomal genome was 3.7 Mb in size with 46% GC content and 3192 protein-coding genes. Additional extensive bioinformatics investigations were carried out involving whole genome sequence assembly and annotation.

## Data Summary

The draft genome sequence of *L. pentosus* krglsrbmofpi2 has been deposited in GenBank; the accession number is GCA_009295675.1 (Bio-Project accession number PRJNA576968 and BioSample accession number SAMN13015363). The NCBI accession number of the 16S rRNA gene sequence of the isolated strain is MN165450 (formerly it was categorized under *Lactobacillus plantarum*). The genome was uploaded under the name *Lactobacillus pentosus*; later, it was renamed *Lactiplantibacillus pentosus*. The strain name is krglsrbmofpi2.

## Announcement

Numerous ethnic groups around the world have a history of integrating fermented foods into their religious and cultural customs [1]. Probiotic bacteria, which can generate organic acids, ethanol and antibiotic compounds, are often present in these foods [2]. Bacterial strains, including of the genera *Lactobacillus*, *Bacillus* and *Bifidobacterium,* comprise most of the strains used in fermentation, while some yeast cultures are also utilized as probiotic cultures [3]. *Lactiplantibacillus pentosus*, formerly known as *Lactobacillus pentosus* and *Lactobacillus plantarum*, is one of the most recent species to have undergone identifiable modifications [4,5]. A comparative genomic investigation of *L. pentosus* and *L. plantarum*, two very closely related species, revealed that * L. pentosus* possesses similar probiotic traits, such as the production of antimicrobial compounds, stress response genes and genes involved in adhesion in the intestinal mucosa. Additionally, *L. pentosus* supports the maintenance of a healthy gut [6]. In the present investigation, the genome of *L. pentosus* strain krglsrbmofpi2 isolated from conventional fermented rice in Himachal Pradesh, India, was sequenced.

Initially, various fermented food samples, including a fermented rice sample, were collected from different locations across Himachal Pradesh. To promote bacterial growth, 100 µl of the fermented rice sample was added to 9.9 ml of peptone broth and incubated at 37 °C for 24 h. After incubation, microbial growth was detected in the peptone broth. To isolate different bacterial strains, the culture was prepared in a series of dilutions. After mixing and vortexing fresh 900 µl De Man–Rogosa–Sharpe (MRS) medium, 100 µl of the culture was added. From this point, fresh MRS was further diluted by adding 100 µl of the diluted culture and this process was repeated until the dilution reached 10^–5^. To isolate different colonies, all dilutions (10^−1^–10^–5^) were spread on MRS agar plates. The plates were incubated at 37 °C for 24 h. Different colonies with different morphological characteristics (size, shape and colour) were selected. To ensure purity, approximately 50 colonies were transferred to fresh MRS agar plates. After extraction, the individual colonies were cultured in MRS broth. The cultures were centrifuged and the supernatants were tested for antibacterial activity against *Salmonella enterica* subsp*. enterica* serovar Typhi after growing in MRS broth for 24 h. One strain that showed remarkable antibacterial activity was selected for further investigation. Fresh MRS broth was spiked with the selected strain, which was then allowed to grow overnight [7]. Glycerol stocks were then prepared by adding 50% glycerol to the culture. These stocks were stored at −20 °C for further studies. The genomic DNA of the strain was purified using the bacterial genomic DNA purification kit (Gene JET; Thermo Fisher Scientific). Whole genome sequencing was done using Illumina Hi Seq 1000 technology (Sandor Speciality Diagnostics), and the resulting sequences were submitted to NCBI (National Center for Biotechnology Information). Genomic DNA was assembled in contigs using the SPAdes v.3.13.1 assembler. Gene predictions were made with RAST Server (Rapid Annotation Utilising Subsystem Technology) using standard parameters (http://RAST.nmpdr.org) [8], and the annotation was implemented by the Prokaryotic Genome Annotation Pipeline (PGAP) at the NCBI [9,11].

**Fig. 1.**
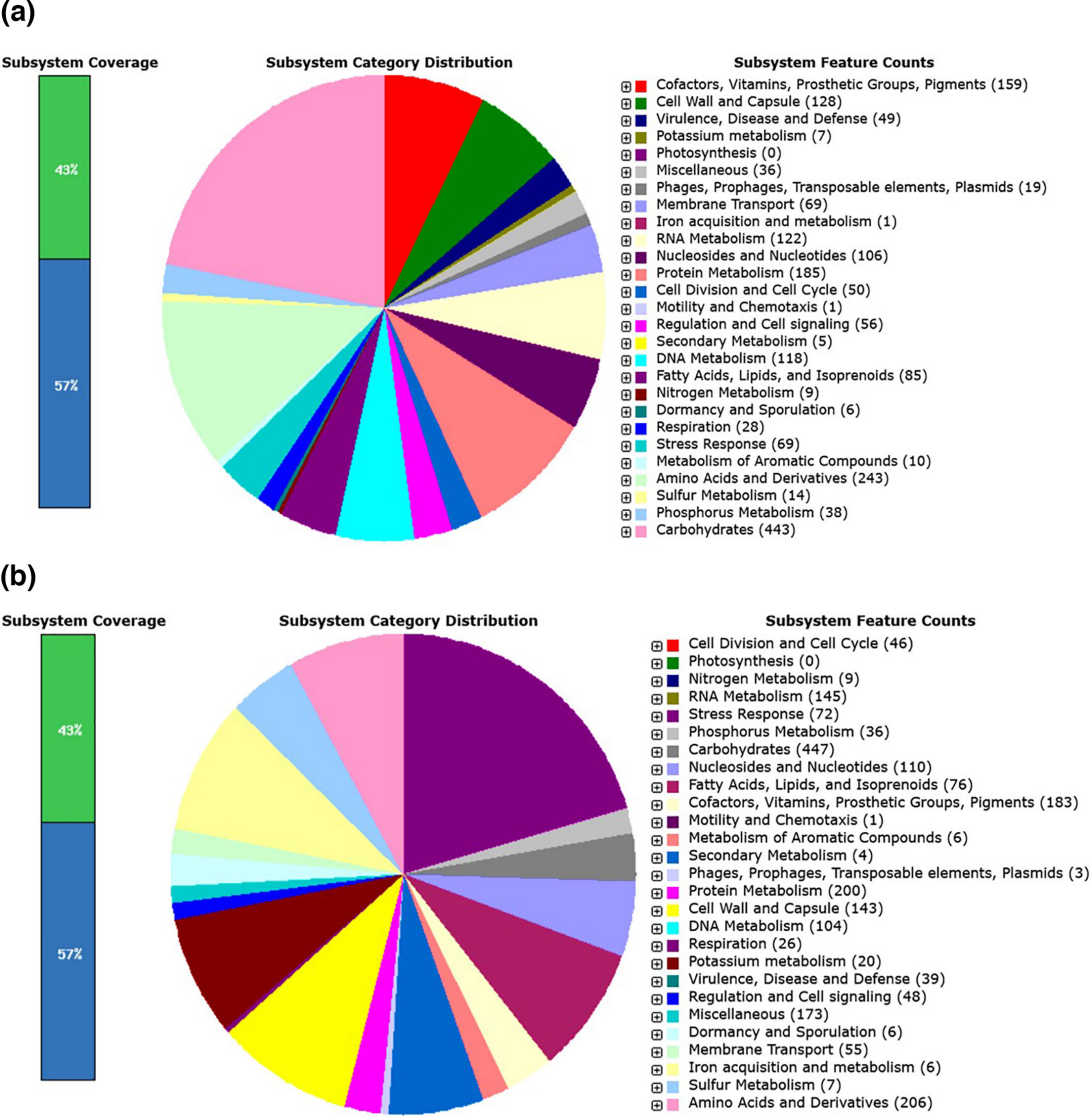
An overview of the subsystem categories assigned to the genomes (RAST annotation). (**a**) The isolated strain, *L. pentosus* krglsrbmofpi2; (**b**) *L. plantarum* WCFS1.

As per genome sequence analysis, the genome is 3.7 Mb, with 46% GC content, an N50 value of 213 kb, an L50 of 7 and 3418 genes, of which 3192 are protein-coding genes. The genome has 55 contigs. Of 76 RNA genes, 65 are tRNA genes, seven are rRNA genes and four are ncRNA (non-coding RNA) genes. The genome also possesses 150 pseudogenes and four CRISPR arrays (GCF_009295675.1, NCBI). Table 1 summarizes the general characteristics of the genome assembly and annotation. RAST annotation reveals 350 subsystems present in the genome of *L. pentosus* krglsrbmofpi2. The 350 subsystems identified represent various functional categories within the genome. For instance, these include metabolic pathways, transport systems, genes regulating gene expression and cellular responses, genes contributing to cell wall and membrane formation, and genes that help the organism survive under stressful conditions. The qualities present in the genome of *L. pentosus* krglsrbmofpi2 contained 43% in subsystem and 57% in non-subsystem inclusion qualities. This indicates that distinct subsystems are related to 43% of the genes in the annotated genome. Depending on which annotation database was used, these genes are associated with established functional categories or pathways. The majority of genes in the genome (57%) have no assigned subsystem. They may not fit into any of the specified subsystems in the database used for annotation, be categorized as hypothetical proteins or have unknown activities [12]. The isolated strain’s closest phylogenetic neighbours were identified by RAST analysis, which also shed light on the strain’s genomic relationships. In particular, the findings showed that the new strain and *Lactobacillus plantarum* WCFS1, which was isolated from human saliva, share many similarities [13]. These findings highlight the genetic proximity and potential functional similarities between the isolated strain and well-characterized *Lactobacillus* spp. (Fig. 1a represents the RAST annotation results of predicted gene distribution in *L. pentosus* krglsrbmofpi2, and Fig. 1b *L. plantarum* WCFS1). The corresponding predicted genes of the isolated *L. pentosus* strain by RAST annotation are shown in Table 2.

**Table 1.** Summary of the general characteristics of the genome assembly and annotation of *L. pentosus* krglsrbmofpi2

Assembly							Annotation			
Genome size (Mb)	Number of scaffolds	Scaffold N50 (kb)	Scaffold L50	Number of contigs	GC content (%)	Genome coverage	Genes	Protein-coding	Non-coding	Genes (RNA)
3.7	51	213	7	55	46	100.0×	3418	3192	76	76

**Table 2.** RAST annotation categories and sub-categories of the *L. pentosus *krglsrbmofpi2 genome

Sl no.	Subsystem category	Subsystem feature counts	Subsystem subcategory
1	Cofactors, vitamins, prosthetic groups, pigments	159	Biotin, Quinone cofactors, Tetrapyrroles, Riboflavin, FMN, FAD, Pyridoxine, NAD and NADP, Folate and pterines, Lipoic acid, Coenzyme A
2	Cell wall and capsule	128	Capsular and extracellular polysaccharides, Gram-positive cell wall components, Cell wall and capsule
3	Virulence, disease and defence	49	Adhesion, Resistance to antibiotics and toxic compounds, Invasion and Intracellular resistance
4	Potassium metabolism	7	Potassium homeostasis
5	Miscellaneous	36	Plant-prokaryote DOE project, Miscellaneous without subcategory
6	Phages, prophages, transposable elements, plasmids	19	Phages, Prophages-phage tail protein, Phage replication, Phage packaging machinery, Phage capsid protein, Phage tail fibre protein
7	Membrane transport	69	ABC transporters, Protein translocation across the cytoplasmic membrane, Cation transporters, Uni-, sym- and antiporters, Energy-coupling factors (ECF) class transporters
8	Iron acquisition and metabolism	1	Encapsulating protein for DyP-type peroxidase and ferritin-like protein oligomers
9	RNA metabolism	122	RNA processing and modification, Transcription, Group II intron-associated genes
10	Nucleosides and nucleotides	106	Pyrimidines, Purines, Detoxification, Ribonucleotide reduction, Hydantoin metabolism, Adenosyl nucleosidases
11	Protein metabolism	185	Protein folding, Protein biosynthesis, Protein processing and modification, Protein degradation
12	Cell division and cell cycle	50	Checkpoint control, Cell division, and Cell cycle – no subcategory, Control of cell elongation – division cycle in Bacilli, Macromolecular synthesis operon, Bacterial cytoskeleton
13	Motility and chemotaxis	1	Bacterial chemotaxis
14	Regulation and cell signalling	56	Programmed cell death and Toxin–antitoxin systems, Regulation, and Cell signalling without subcategory – cAMP signalling in bacteria, LysR-family proteins in *Salmonella enterica* Typhimurium, LysR-family proteins in *Escherichia coli*, HPr catabolite repression system, Sex pheromones in *Enterococcus faecalis* and other *Firmicutes*, Stringent response, Cell envelope-associated LytR – CpsA – Psr transcriptional attenuators
15	Secondary metabolism	5	Lanthionine synthetases, Auxin biosynthesis
16	DNA metabolism	118	DNA repair, CRISPRs, DNA metabolism, DNA replication, DNA recombination, DNA uptake, Competence
17	Fatty acids, lipids and isoprenoids	85	Phospholipids, Triacylglycerols, Fatty acids, Isoprenoids
18	Nitrogen metabolism	9	Nitrate and nitrite ammonification, Denitrifying reductase gene clusters
19	Dormancy and sporulation	6	Sporulation-associated proteins with broader functions, Sporulation cluster
20	Respiration	28	Biotin, Electron accepting reactions, Electron donating reactions, Sodium ion-coupled energetics
21	Stress response	69	Osmotic stress, Oxidative stress, Cold shock, Heat shock, Detoxification
22	Metabolism of aromatic compounds	10	Peripheral pathways for catabolism of aromatic compounds, Aromatic amine catabolism
23	Amino acids and derivatives	243	Glutamine, glutamate, aspartate and asparagine biosynthesis, Ammonia assimilation, Histidine metabolism, Arginine, Urea cycle, polyamines, Lysine, threonine, methionine and cysteine, Creatine, and Creatinine degradation, Branched-chain amino acids, Aromatic amino acids, and derivatives, Proline and 4-hydroxyproline, Alanine, serine and glycine biosynthesis, Glycine and serine utilization
24	Sulphur metabolism	14	Organic sulphur assimilation, Thioredoxin-disulphide reductase, Galactosyl ceramide, Sulphatide metabolism
25	Phosphorus metabolism	38	High-affinity phosphate transporter and control of PHO regulon, Phosphate metabolism, Polyphosphate, and Phosphonate metabolism
26	Carbohydrates	443	Central carbohydrate metabolism, Amino sugars, Di- and oligosaccharides, One-carbon metabolism, Organic acids, Fermentation, Sugar alcohols, Polysaccharides, and Monosaccharides

CpsAcapsular polysaccharide expression regulatorCRISPRsclustered regularly interspaced short palindromic repeatsDOEDepartment of EnergyDyPdye decoloring peroxidasesFADFlavin adenine dinucleotideFMNFlavin mononucleotideHPrhistidine-phosphorylatable phosphocarrier proteinLytRlytic repressorNADnicotinamide adenine dinucleotideNADPnicotinamide adenine dinucleotide phosphatePHO regulonphosphate regulonPsrPBP 5 synthesis repressor

Other strains of *L. pentosus*, such as *L. pentosus* SLC13 from mustard pickles [14] and *L. pentosus* KCA1 isolated from a pre-menopausal woman’s vagina [15], are also known to have similar properties. It is also comparable with the recent studies of López-García *et al*. [16]. They presented a comprehensive *in silico* analysis of the *L. pentosus* strain LPG1 isolated from table olive biofilms. The complete genome of strain LPG1 consisted of 3  700  533 bp with a G+C content of 46.23%. This strain had a circular chromosome of 3  619  252 bp, a G+C content of 46.34% and two sequenced plasmids [16]. Notably, *L. pentosus* krglsrbmofpi2 represents the only Indian *L. pentosus* genome with a complete sequence submitted to NCBI among the 104 genomes in the NCBI database (https://www.ncbi.nlm.nih.gov/datasets/genome/?taxon=1589). Based on the RAST annotation results, the Indian * L. pentosus* isolate demonstrates a range of functional attributes that suggest its potential as a functional food with probiotic qualities. The annotation has identified key metabolic pathways and gene clusters that indicate its ability to contribute beneficial effects in a food matrix. Detailed functional studies are underway to validate these preliminary findings and further elucidate the strain’s potential health benefits.

## Abbreviations

CRISPRs: clustered regularly interspaced short palindromic repeats
MRS: Man–Rogosa–Sharpe
NCBI: National Center for Biotechnology Information
RAST: rapid annotation utilising subsystem technology
